# A Study on the Entire Take-Over Process-Based Emergency Obstacle Avoidance Behavior

**DOI:** 10.3390/ijerph20043069

**Published:** 2023-02-09

**Authors:** Yi Li, Zhaoze Xuan, Xianyu Li

**Affiliations:** 1 Logistics Research Center, Shanghai Maritime University, Shanghai 201306, China; 2 Tongji Architectural Design (Group) Co., Ltd., Shanghai 200092, China; 3 Shanghai Research Center for Smart Mobility and Road Safety, Shanghai 200092, China

**Keywords:** automated driving, take-over behavior, take-over process, scenario urgency

## Abstract

Nowadays, conditional automated driving vehicles still need drivers to take-over in the scenarios such as emergency hazard events or driving environments beyond the system’s control. This study aimed to explore the changing trend of the drivers’ takeover behavior under the influence of traffic density and take-over budget time for the entire take-over process in emergency obstacle avoidance scenarios. In the driving simulator, a 2 × 2 factorial design was adopted, including two traffic densities (high density and low density) and two kinds of take-over budget time (3 s and 5 s). A total of 40 drivers were recruited, and each driver was required to complete four simulation experiments. The driver’s take-over process was divided into three phases, including the reaction phase, control phase, and recovery phase. Time parameters, dynamics parameters, and operation parameters were collected for each take-over phase in different obstacle avoidance scenarios. This study analyzed the variability of traffic density and take-over budget time with take-over time, lateral behavior, and longitudinal behavior. The results showed that in the reaction phase, the driver’s reaction time became shorter as the scenario urgency increased. In the control phase, the steering wheel reversal rate, lateral deviation rate, braking rate, average speed, and takeover time were significantly different at different urgency levels. In the recovery phase, the average speed, accelerating rate, and take-over time differed significantly at different urgency levels. For the entire take-over process, the entire take-over time increased with the increase in urgency. The lateral take-over behavior tended to be aggressive first and then became defensive, and the longitudinal take-over behavior was defensive with the increase in urgency. The findings will provide theoretical and methodological support for the improvement of take-over behavior assistance in emergency take-over scenarios. It will also be helpful to optimize the human-machine interaction system.

## 1. Introduction

Automated driving technology is considered to be an effective way to reduce traffic accident rates. Traditional automobile companies have released automated driving systems with similar functionality and have set up various road zones for conducting tests of real-world scenarios [1]. Fully automated driving vehicles have significant advantages in terms of efficiency, safety, and energy consumption. However, most of the automated driving vehicles on the market are at L2 and L3 levels, which are not yet capable of fully automated driving in real-world road environments. Therefore, manual human driving is still required when the driving environment’s complexity exceeds the system’s preset function or the system fails. In the December 2015 report on automated driving testing published by Google, a total of 272 instances were reported to have occurred during automated driving tests in which the automated driving vehicles immediately transferred control to the driver due to the failure of the automatic detection technology [2]. Hence, human-machine co-driving serves as a transition phase from the manual driving state to the fully automated driving state, which will continue for a long time in the future. Drivers’ improper take-over behavior will lead to traffic accidents. To solve this problem, the study of take-over behavior differences is important to improve the safety and stability of automated driving systems. Many researchers have conducted studies on the take-over process and the factors influencing take-over behavior.

### 1.1. Process of Take-Over

Lu classified the control switching of automated driving vehicles into three categories based on the initiator and compulsion of switching, including human-initiated optional switching, human-initiated compulsory switching, and system-initiated compulsory switching [3]. This study focused on the take-over process under the system-initiated compulsory switching. Currently, there is no clear definition of the beginning and end of the take-over process. Gold divided the take-over process into four phases. Among them, the time when the driver put their hands and feet back on the steering wheel and brake pedal was defined as the moment when the take-over begins; the moment when the driver took control of the vehicle by pressing the brake pedal or turning the steering wheel was defined as the moment when the take-over ends [4]. However, different take-over behaviors lead to different take-over processes. For example, most of the take-over behavior in the following scenario is braking, and the moment of take-over only needs to focus on the brake pedal. Nevertheless, the take-over behavior in the obstacle avoidance scenario includes braking and steering, and the moment of take-over needs to consider the steering wheel and brake pedal together. Zeeb defined the point of a successful driver take-over as the moment when the steering wheel angle exceeds 2° or the brake pedal position exceeds 10% through extensive experiments [5].

### 1.2. Influencing Factors

#### 1.2.1. Influence of HMI

The HMI is the medium through which the driver interacts with the automated driving system. Proper interaction design facilitates better driver take-over behavior. Current HMI is generally issued in the form of session boxes (visual), voice (auditory), vibration (tactile), or combined modes [6,7,8,9]. Different interaction methods have a certain degree of influence on the driver’s take-over effect. In emergency take-over scenarios, prompts that help the driver react quickly are most appropriate. Forster found that auditory prompts require shorter reaction times than visual prompts [10]. Meanwhile, different words can convey different levels of urgency. For example, Bazilinskyy found that faster speech rates make drivers feel more urgent and commanding than slower speech rates [11]. Some short utterances, such as “Danger, take over” generate a higher subjective sense of urgency than “Please take over” [11]. A high-urgency voice style produces a higher subjective sense of urgency than a monotonous voice style [12].

The take-over budget time is an important safety indicator in the design of HMI. The take-over budget time is defined as the moment from the prompt of the automated driving system to the system limit [13]. A reasonable take-over budget time allows the driver to take better control of the vehicle. Eriksson and Stanton reviewed 25 papers and found the most frequently used time budgets were 3 s, 4 s, 6 s, and 7 s [14]. Different take-over budget times give different time pressures, forcing the driver to take over differently. Gold studied the take-over performance derived from take-over budget time scenarios of 5 s and 7 s and found that the shorter the take-over budget time, the faster the reaction response, while the shorter the budget time, the poorer the quality of take-over behavior [15].

However, the time required for the driver to take over varies greatly due to the driver’s characteristics, non-driving-related tasks, driving environment, and so on. In scenarios without time pressure, the mean take-over time was in the range of 4.5 to 6.0 s, but the actual range was 1.9~25.7 s [14]. In contrast, in scenarios with time pressure, the take-over time was mostly in the range of 3~7 s [16,17]. Lin studied the take-over performance in emergency take-over scenarios with a take-over budget time of 3 s, 4 s, and 5 s and found that the reaction time for drivers (no task) ranged from 0.8 to 1.8 s [18].

#### 1.2.2. Influence of Traffic Environment

The traffic environment is the main factor that drivers need to focus on during the take-over process. The complexity of the traffic environment largely affects the driver’s take-over behavior. Gold’s study found that complex traffic densities led to longer take-over times and poorer take-over quality [4]. When the driver is in an emergency avoidance scenario, he will pay less attention to the HMI and more attention to the surrounding road environment. Du found an interaction influence between traffic density and cognitive load, meaning that when traffic density was high, the driver’s attention would be more focused on the road to improve situational awareness [19].

Meanwhile, the dynamics of the surrounding vehicles force drivers to take some more conservative actions in high-density traffic scenarios. For example, using steering or acceleration operations cautiously results in increased braking frequency for drivers [20,21,22]. At the same time, the reduced collision time between vehicles due to the smaller travel spacing also increases the probability of collision during take-over to some extent, increasing the probability of collision for drivers in high-density scenarios by about 40% compared to low-density conditions [23].

### 1.3. Urgency of Scenarios

Take-over behavior under conditional automated driving is generally divided into two types. The first situation is a non-emergency scenario. For example, when entering an area that is not suitable for automated driving, the automated driving system initiates the take-over or the driver initiates the take-over. In this type of situation, the driver has plenty of time to take over. The second situation is an emergency scenario. For example, if the traffic environment is too complex for the operational design domain or the vehicle detection system fails, a mandatory take-over will be initiated by the automated driving system. In this type of scenario, the driver needs to complete the driving task within a limited time. Scenarios with different levels of urgency lead to different take-over behaviors. Lin defined the takeover urgency as the take-over budget time and found that the take-over urgency (take-over budget time of 3, 4, 5 s) has no effect on the reaction time; however, the synthetic acceleration under the take-over budget time of 3, 4 s increases significantly compared to the 5 s’s [18]. Wu created different levels of urgency by constructing different take-over scenarios, including other vehicles inserting into the current lane (semi-urgent), avoiding an obstacle vehicle ahead (urgent), and avoiding an obstacle vehicle in a shorter time (urgent) [24]. Currently, the consideration of take-over scenario urgency is basically from the time dimension or changing the traffic environment. In this study, the take-over budget time and traffic density were chosen to define the scenario urgency by considering the time factor and the traffic environment factor.

In summary, previous studies have been more comprehensive in studying the changes in driver take-over behavior by constructing different take-over scenarios and setting different interference factors. They evaluated take-over behavior mainly by instantaneous indicators, such as reaction time and maximum acceleration, etc.; the take-over process mainly started from the system prompting to take over to the driver’s stable control of the vehicle [14,25,26]. However, when drivers are asked to take over in high-risk scenarios, especially obstacle avoidance scenarios that require both lateral and longitudinal control by the driver, panic and impatience can occur. Even if the driver stabilizes the vehicle in a short time, the panic will continue to affect the driver’s driving ability. At the same time, the influence of the traffic environment on the driver is further enhanced as the driver’s take-over ability deteriorates. Therefore, this study designs a 2 × 2 factorial simulation experiment in an emergency obstacle avoidance scenario, while extending the take-over process to the moment when the driver switches the vehicle back to the automated driving state. By collecting the dynamics parameters and the driver’s operation parameters during the entire take-over process, the effect of different traffic densities and take-over budget times on the driver’s take-over process is explored. This study provides new insights and support for the design of automated driving systems in the upcoming era of human-machine co-driving.

## 2. Methods

### 2.1. Equipment

**Figure 1:**
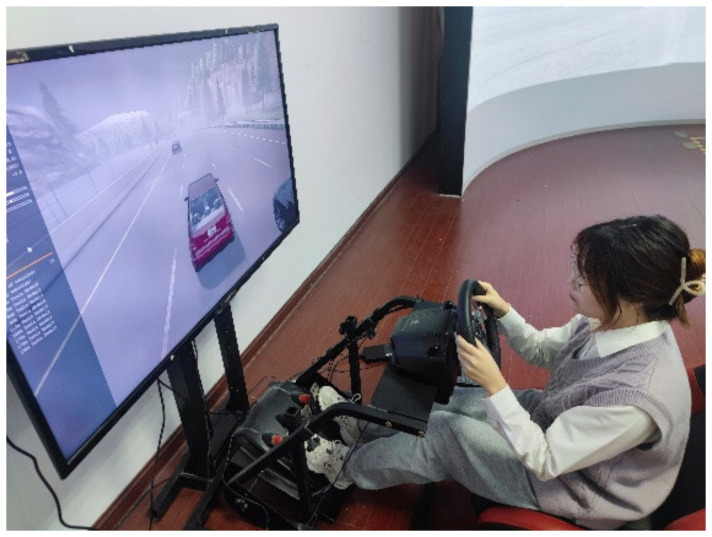
Driving simulation equipment.

The experiment equipment consists mainly of a set of driving simulators and CARLA 0.9.13 (open-source automated driving simulation software). The hardware of the driving simulator includes a Logitech G27 steering wheel, a gas pedal, a brake pedal, a computer, and a monitor. The driving simulation software supports automated driving simulation, traffic scenario construction, traffic flow generation, and other functions. In addition, various data can be collected by a variety of sensor suites for automated driving, including vehicle coordinates, vehicle speed, acceleration, pedal position, steering wheel angle, etc. (see Figure 1).

### 2.2. Participants

A total of 40 drivers (20 male and 20 female) participated in this experiment. Considering that young people are more receptive to emerging technologies, the participants included university students as well as university teachers. Their ages ranged from 22 to 37 years old (AVG. = 26.25, S.D. = 3.01). Before the experiment, each driver was in good health and had normal vision.

### 2.3. Experiment Design

#### 2.3.1. Environment Parameters

This study used traffic density and take-over budget time to measure the urgency of take-over. Gold classified the traffic density into 0, 10, and 20 vehicles per kilometer when studying the effect of traffic density on take-over behavior [15]. In order to distinguish the urgency of each scene more clearly, this study only divided the traffic density into high and low dimensions. A traffic density of less than 20 vehicles per kilometer is recorded as low-density traffic, and a traffic density greater than 20 vehicles per kilometer is recorded as high-density traffic in this study. In terms of take-over budget time, in order for the driver to feel a clear time pressure, this study set the take-over budget time to 3 s and 5 s (Kathrin Zeeb et al., 2015; Marcel Walch et al., 2015) [5,27].

**Figure 2:**
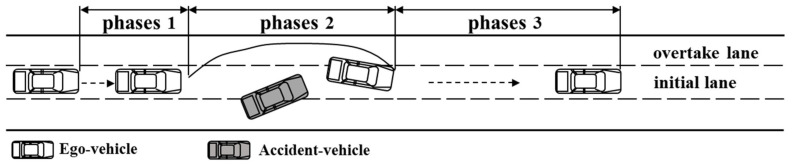
Schematic diagram of the entire process of taking over.

The entire take-over process begins the moment the driver presses the switch button and ends the moment the driver switches back to automated driving. Based on Gold’s take-over process analysis [4], the entire take-over process was divided into three phases, as shown in Figure 2.

Phase 1: Reaction phase. After the driver received a sudden take-over prompt, he or she needed to be cognizant of the take-over scenario before performing the corresponding take-over operation. In this study, this phase was defined as the system issuing a take-over prompt until the driver presses the toggle button.

Phase 2: Control phase. The control phase is the main phase in which the driver takes over. The driver would adopt the corresponding lateral and longitudinal control behaviors based on his or her judgment of the surrounding environment. This study defined this phase as the time from when the driver pressed the take-over button until the center of the vehicle entered the initial lane after the vehicle completed the avoidance operation.

Phase 3: Recovery phase. After the driver experienced the emergency scenario of taking over, the physiology and psychology would be subject to a few fluctuations in a short time, affecting subsequent driving. In this study, this phase was defined as after the vehicle center entered the initial lane until the driver switched back to automated driving.

In the simulated driving process, different take-over phases were distinguished according to the system prompt moment, driver switch moment, and vehicle body position. At the same time, Carla recorded operation parameters such as throttle operation, steering wheel turn, and brake pedal operation, as well as vehicle dynamics parameters such as average speed and lateral deviation at 60 Hz. Each parameter is defined as shown in Table 1.

#### 2.3.2. Take-Over Scenarios

**Figure 3:**
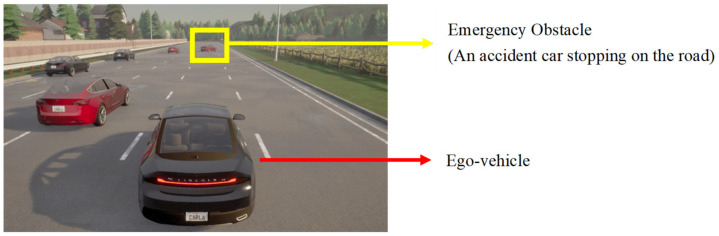
Emergency obstacle avoidance simulation scenario.

The driving scenario relied on Carla to create a highway. The highway was a one-way, five-lane highway, with each lane being 3.5 m wide. The simulated scenario generated a random traffic flow ranging from 0 to 30 vehicles/km, with the speed of the random traffic flow set at 60 km/h. Assuming that the automated driving vehicle was always cruising in the second lane at 88 km/h, the vehicle needed to be switched from automated driving to manual driving upon receiving a prompt from the system to circumvent the obstacle vehicle in a collision-free status, as shown in Figure 3. A take-over prompt was issued by voice when the vehicle arrived at the designated location. The speaker broadcasted a female voice: “Danger, take over”.

The obstacle vehicle was placed diagonally in the first and second lanes at an angle of 25 degrees from the road centerline. The driver did not need to operate during the automated driving process but only needed to control the gas pedal, brake pedal, and steering wheel after the system prompted him to take over.

**Figure 4:**
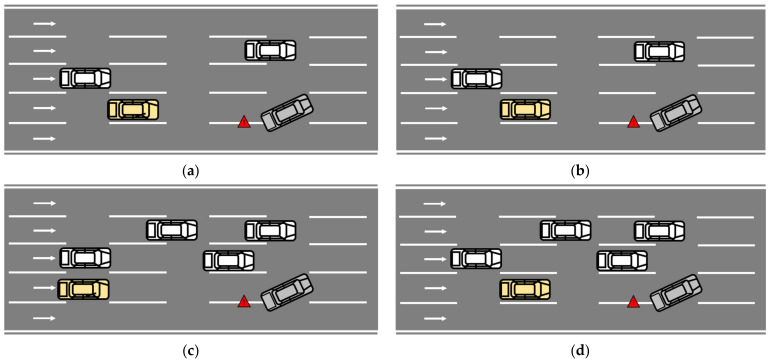
Take-over scenarios with different urgency. (**a**) Scenario 1; (**b**) Scenario 2; (**c**) Scenario 3; (**d**) Scenario 4. White vehicles are manual human driving vehicles; yellow vehicle is automated driving vehicle; gray vehicle is accident vehicle.

In order to avoid the problem that the number of vehicles around the obstacle vehicle varies too much under the same traffic density or not much under different traffic densities, this study divided the traffic density by the real-time number of vehicles within five hundred meters in front of and behind the obstacle car instead of dividing the traffic density by all the vehicles within the whole simulation environment. Figure 4 shows the take-over scenario with different urgency levels. In Scenario 1, the experiment results showed low traffic density (<20 pcu/km) and a take-over budget time of 5 s, in which the scenario’s urgency is considered to be the lowest. In Scenario 2, the experiment results with low traffic density (<20 pcu/km) and a take-over budget time of 3 s were recorded. In Scenario 3, the experiment results showed high traffic density (>20 pcu/km) and a take-over budget time of 5 s was recorded. In these two scenarios, the scenario’s urgency is considered to have somewhat increased. In Scenario 4, the experiment results showed high traffic density (>20 pcu/km) and a take-over budget time of 3 s, in which the scenario urgency is considered to be the highest.

### 2.4. Simulation Procedure

The experiment steps were as follows.

Step 1: Before the experiment, the driver needed to perform the driving practice for 5 to 10 min in the simulation environment until the driver was familiar with the apparatus.

Step 2: In the normal simulation, the driver needed to face 2 × 2 emergency take-over scenarios. Before receiving the system take-over prompt, the driver did not need to observe the surrounding traffic environment without driving operations; after receiving the system take-over prompt, the driver must switch the vehicle state by pressing the switch button and then manually control the vehicle to avoid the obstacle vehicle.

Step 3: After avoiding the obstacle vehicle, the driver still needed to control the vehicle to move back to the initial lane. When the driver has finished the lane-changing behavior, he/she must immediately switch back to the automated driving state.

All participants should repeat the above process until all four scenarios have been tested. If a collision occurred during the experiment, the experiment would be terminated and the corresponding data would be deleted. The position of each obstacle vehicle was set randomly in order to prevent the learning effect. The whole driving process lasted about 8 min.

## 3. Results

### 3.1. Analysis of Lateral Take-Over Parameters

The lateral take-over parameters collected in this study include: steering wheel reversal rate, maximum steering angle, lateral deviation, and lateral deviation rate.

#### 3.1.1. Variation Pattern of Operation Parameters in Each Phase

**Figure 5:**
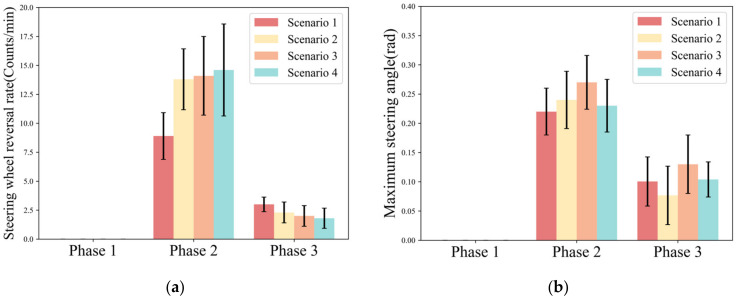
Variation pattern of lateral operation parameters. (**a**) Steering wheel reversal rate; (**b**) Maximum steering angle.

The steering wheel reversal rate reflects how much the driver relies on the lateral control of the vehicle. Maximum steering angle reflects the driver’s aggressive level of lateral control of the vehicle, which characterizes the risk of driver take-over. Figure 5 shows the steering wheel reversal rate, and the maximum steering angle for each phase of the driver in different scenarios. Phase 1 is the driver’s reaction phase, during which the driver had no steering wheel control. The driver’s control of the steering wheel was mainly concentrated in Phase 2. It can be seen from Phase 2 that the steering wheel reversal rate in Scenario 1 (AVG. = 8.9) was significantly lower than the other three scenarios (Scenario 2: AVG. = 13.8; Scenario 3: AVG. = 14.1; Scenario 4: AVG. = 14.6). Meanwhile, the higher the scenario urgency, the greater the standard deviation of the steering wheel reversal rate (Scenario 1: S.D. = 2.02; Scenario 2: S.D. = 2.63; Scenario 3: S.D. = 3.44; Scenario 4: S.D. = 3.97). The maximum steering angle showed a trend of “increasing-decreasing” (Scenario 1: AVG. = 0.22; Scenario 2: AVG. = 0.24; Scenario 3: AVG. = 0.27; Scenario 4: AVG. = 0.23). It can be seen from Phase 3 that the steering wheel reversal rate declined from Scenario 1 to Scenario 4 (Scenario 1: AVG. = 3; Scenario 2: AVG. = 2.3; Scenario 3: AVG. = 2; Scenario 4: AVG. = 1.8). The maximum steering angle in different urgency scenarios shows a discrete trend.

Table 2 shows the statistical results of the steering wheel reversal rate and the maximum steering angle under different urgency scenarios. The results show that both take-over budget time (F = 6.767, *p* = 0.013) and traffic density (F = 8.354, *p* = 0.006) have a significant effect on steering wheel reversal rate in Phase 2. Two influencing factors have an interaction effect on the steering wheel reversal rate in Phase 2. In Phase 3, there is a significant difference in steering wheel slew rate at different traffic densities (F = 7.31, *p* = 0.01). In addition, there is no significant difference in the maximum steering angle with different traffic densities and take-over budget times in Phase 2 and Phase 3.

#### 3.1.2. Variation Pattern of Dynamics Parameters in Each Phase

**Figure 6:**
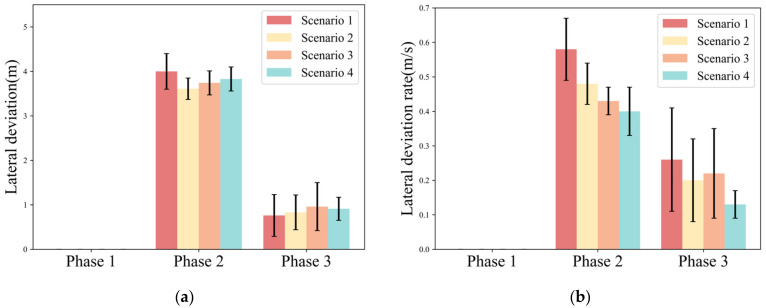
Variation pattern of lateral dynamics parameters. (**a**) Lateral deviation; (**b**) Lateral deviation rate.

Lateral deviation reflects the fluctuation of the lateral position of the vehicle and can characterize the lane departure risk. The lateral deviation rate reflects the rate of change of the vehicle’s lateral deviation with time. Figure 6 illustrates the lateral deviation and lateral deviation rate for each phase of the driver in different scenarios.

Phase 1 is still with the automated driving system controlling the vehicle, and no lateral deviation of the vehicle is detected.

Phase 2 is the key phase for the driver to avoid the obstacle vehicle, so this study focuses on the lateral deviation in this phase. In Figure 6a, the lateral deviation presented no major difference among the four scenarios (AVG. = 3.61~4.01). However, the standard deviation of the driver’s lateral deviation in Scenario 1 (S.D. = 0.405) was significantly larger than that of the other scenarios (Scenario 2: S.D. = 0.246; Scenario 3: S.D. = 0.278; Scenario 4: S.D. = 0.278). As seen in Figure 6b, the lateral deviation rate under different take-over scenarios has a clear trend of change. When the scenario urgency is low, the driver’s lateral deviation rate is higher (Scenario 1: AVG. = 0.573). When the urgency was higher, the lateral deviation rate of the drivers was lower (Scenario 4: AVG. = 0.405).

In Phase 3, there is no significant change in lateral deviation among the four scenarios (AVG. = 0.76~0.96). The trend of the lateral deviation rate is similar to that of Phase 2. The lateral deviation rate decreases with the increase in urgency. Meanwhile, the standard deviation of the lateral deviation rate under Scenario 4 is significantly smaller than that of the other three scenarios.

Table 3 shows the results of the statistical analysis of the lateral deviation and the lateral deviation rate under different urgency scenarios. The results show that there is a significant interaction between take-over budget time and traffic density on the lateral deviation in Phase 2 (F = 5.708, *p* = 0.022). Both traffic density (F = 23.269, *p* < 0.001) and take-over budget time (F = 7.710, *p* = 0.008) exhibit a significant effect on lateral deviation rate in Phase 2. In Phase 3, there is no significant difference between lateral deviation and lateral deviation rate with traffic density and take-over budget time.

### 3.2. Analysis of Longitudinal Take-Over Parameters

The longitudinal take-over parameters collected in this study include average speed, braking rate, and accelerating rate.

#### 3.2.1. Dynamics Parameters in Each Phase

**Figure 7:**
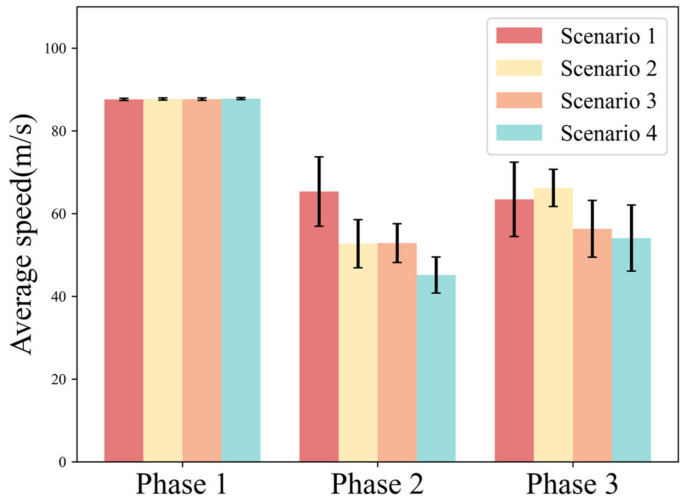
Variation pattern of average speed.

The average speed reflects the stability and safety of the longitudinal motion of the vehicle. Figure 7 shows the average speed of the driver in each phase under different scenarios.

In Phase 1, the average speed in the four scenarios is almost unchanged. In Phase 2, Scenario 1 has the highest average speed (AVG. = 65.34, S.D. = 8.39); Scenario 2 (AVG. = 52.72, S.D. = 5.83) and Scenario 3 (AVG. = 52.87, S.D. = 4.69) have similar average speeds; Scenario 4 has the lowest average speed (AVG. = 45.15, S.D. = 4.35). In Phase 3, the average speeds of Scenario 1 (AVG. = 63.43, S.D. = 8.99) and Scenario 2 (AVG. = 66.21, S.D. = 4.51) are closer, while Scenario 3 (AVG. = 56.30, S.D. = 6.86) and Scenario 4 (AVG. = 54.08, S.D. = 7.98) are closer. Overall, the average speed is higher in scenarios with lower urgency and lower in scenarios with higher urgency.

Table 4 shows the results of the statistical analysis of the average speed under different urgency scenarios. The results show that traffic density and take-over budget time have a significant effect on the average speed in Phase 2. In Phase 3, traffic density has a significant effect on the average speed, but take-over budget time does not have a significant effect on it.

#### 3.2.2. Operation Parameters in Each Phase

**Figure 8:**
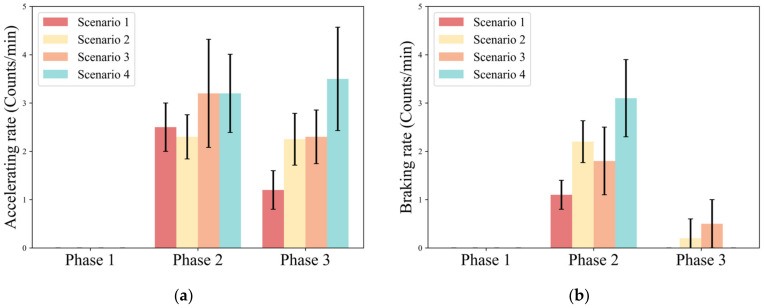
Variation pattern of longitudinal operation parameters. (**a**) Accelerating rate; (**b**) Braking rate.

The acceleration rate reflects the driver’s longitudinal acceleration behavior, which characterizes the safety of the driver’s control of the vehicle. Braking rate reflects the driver’s longitudinal deceleration behavior, which characterizes the stability of the driver’s control of the vehicle. Figure 8 shows the number of brakes and accelerations in each phase in different scenarios.

As seen in Phase 2, the accelerating rate under Scenario 1 (AVG. = 2.5) and Scenario 2 (AVG. = 2.3) is significantly smaller than that under Scenario 3 (AVG. = 3.2) and Scenario 4 (AVG. = 3.2). Furthermore, the standard deviation of the accelerating rate under Scenario 1 (S.D. = 0.5) and Scenario 2 (S.D. = 0.46) is significantly smaller than that under Scenario 3 (S.D. = 1.12) and Scenario 4 (S.D. = 0.81). The braking rate increased with urgency: 1.1 counts/min for a low-urgency scenario (Scenario 1), 2.2 counts/min and 1.8 counts/min for medium-urgency scenarios (Scenario 3 and Scenario 4), and 3.1 counts/min for a high-urgency scenario (Scenario 4).

In Phase 3, the accelerating rate increases with urgency (Scenario 1: AVG. = 1.2; Scenario 2: AVG. = 2.3; Scenario 3: AVG. = 2.2; Scenario 4: AVG. = 3.6). However, braking behavior is almost absent in Phase 3, and the mean value of the braking rate in Scenario 3 is only 0.45 counts/min.

Table 5 shows the results of the statistical analysis of the accelerating rate and the braking rate under different urgency scenarios. The results show that traffic density shows a significant main effect for accelerating rate (F = 8.347, *p* = 0.006) and braking rate (F = 15.36, *p* < 0.001) in Phase 2. Meanwhile, take-over budget time shows a significant main effect on braking rate (F = 34.56, *p* < 0.001). In Phase 3, both traffic density and take-over budget time show a significant main effect for acceleration rate.

### 3.3. Analysis of Take-Over Time in Each Phase

**Figure 9:**
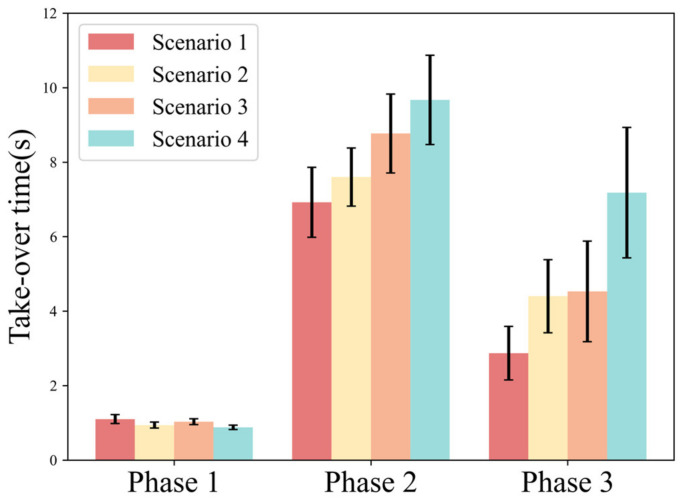
Variation pattern of take-over time.

In Phase 1, reaction time (Scenario 1: AVG. = 1.095; Scenario 2: AVG. = 0.94; Scenario 3: AVG. = 1.033; Scenario 4: AVG. = 0.882) tends to decrease overall as the urgency increases. In Phase 2, control time increases with increasing urgency (Scenario 1: AVG. = 6.918; Scenario 2: AVG. = 7.6; Scenario 3: AVG. = 8.768; Scenario 4: AVG. = 9.667). In Phase 3, the recovery time has the same trend as the control time (Scenario 1: AVG. = 2.873; Scenario 2: AVG. = 4.443; Scenario 3: AVG. = 4.537; Scenario 4: AVG. = 7.189) (Figure 9).

Table 6 shows the results of the statistical analysis of the take-over time under different urgency scenarios. The results show that: take-over budget time has a significant main effect on the response time in Phase 1. Traffic density has a significant main effect on the control time in Phase 2. Both take-over budget time and traffic density have a significant main effect on the recovery time in Phase 3.

## 4. Discussion

In this study, the driver’s take-over time, lateral take-over behavior, and longitudinal take-over behavior under different phases of the emergency obstacle avoidance scenario were investigated, considering the transient effect of take-over budget time and the continuous effect of traffic density.

### 4.1. Influence of Time Budget and Traffic Density on Take-Over Time

The entire take-over time increases when the scenario’s urgency increases. The reason is that the drivers need to spend more time controlling the vehicle due to the higher difficulty of taking over in emergency scenarios. On the other hand, the automated driving system makes the driver suddenly exposed to a high-risk scenario, which would reduce the driver’s trust and lead to a longer manual driving time [28]. This is particularly evident in the driver’s recovery phase.

Reaction time is also an important parameter for studying take-over behavior [29]. It is found that the reaction time decreased with the increasing scenario urgency in this study. Particularly, take-over budget time has a significant effect on reaction time (F = 24.694, *p* < 0.001). This is consistent with the conclusions reached by Gold [15]. However, the faster the driver’s reaction is accompanied by a more intense operation of the vehicle and a relatively poorer quality of take-over. This is caused by the fact that the driver does not have enough time to be aware of the surrounding traffic environment and make the right decisions [30]. Therefore, creating a longer take-over budget time can make it safer for drivers to take over in the face of emergency scenarios when designing an automated driving system.

### 4.2. Influence of Time Budget and Traffic Density on Lateral Behavior

For lateral behavior, the steering wheel reversal rate and lateral deviation rate were the two significant indicators during the control phase. Although the steering wheel reversal rate and lateral deviation rate showed a significant trend with scenario urgency, lateral deviation did not change significantly (M is in the range of 3.61 to 4.01). This is an overcompensation behavior, meaning that the driver compensates for the adverse effects of urgency on driving by increasing the steering wheel correction frequency [31]. There was no significant relationship between maximum steering angle and urgency. However, as seen in Figure 5b, as the urgency of the take-over scenario increases, drivers will adopt a more aggressive lateral deflection behavior in the emergency obstacle avoidance scenario. However, when the take-over scenario is too complex, drivers will make more defensive lateral operations. In summary, drivers in emergency obstacle avoidance scenarios will quickly avoid major risks by increasing the frequency and magnitude of steering wheel corrections. However, when the take-over scenario is too complex, drivers will still increase the steering wheel correction frequency but reduce the correction magnitude at the same time.

During the recovery phase of take-over, the steering wheel reversal rate and lateral deviation are almost constant. However, the lateral deviation rate still decreases with increasing urgency. This indicates that as the scenario urgency increases, drivers take more defensive lane changes even if they have completed the main risk avoidance (avoiding the obstacle vehicle). One reason for this may also be the continuous influence of surrounding vehicles on the driver’s lateral take-over behavior.

### 4.3. Influence of Time Budget and Traffic Density on Longitudinal Behavior

For longitudinal behavior, the braking rating increases with urgency during the control phase. This is consistent with Harbluk’s study, which indicates there are more occurrences of hard braking during the difficult take-over scenario [32]. The accelerating rate is significantly correlated with traffic density and take-over budget time. This is due to multiple hard brakes resulting in lower vehicle speeds. Considering that the driving scenario is on a highway and the traffic speed is high, drivers are forced to increase their speed to avoid adverse effects on the traffic flow due to the pressure of the traffic density during obstacle avoidance.

During the recovery phase, the drivers have almost no braking behavior, indicating that they have a good grasp of the longitudinal control of the vehicle after avoiding the main risks.

## 5. Conclusions

In this study, we constructed a take-over behavior simulation experiment based on an emergency obstacle avoidance scenario and explored the take-over characteristics under different take-over phases in the face of different traffic densities and take-over budget times. More specifically:

1. In terms of lateral take-over behavior, steering wheel reversal rate, and lateral deviation rate are the key parameters in an obstacle avoidance scenario. An appropriate sense of urgency causes drivers to adopt more aggressive steering behavior. An excessive sense of urgency causes drivers to adopt more defensive steering behavior.
2. In terms of longitudinal take-over behavior, drivers will hit the gas and brake pedals frequently in the face of an emergency take-over scenario to make the vehicle reach a stable and safe speed. Meanwhile, the driver’s braking rate is significantly reduced after avoiding the main risk.
3. In terms of take-over time, the higher the scenario urgency, the shorter the driver’s reaction time, the longer the control time and recovery time, and the longer the entire take-over time.

This study still has some limitations. This study creates different traffic densities by controlling the number of vehicles from a macroscopic point of view, but the local traffic densities of each take-over phase are not defined from a microscopic point of view. Furthermore, this study only considers the influence of the traffic environment and human-machine interaction system on the take-over behavior and does not consider the influence of the driver’s personal attributes, such as driving style and age, on the take-over behavior. The next step is to classify the driving styles of drivers and explore the differences in take-over behavior of drivers with different driving styles in the face of emergency take-over scenarios. These findings can provide theoretical and methodological support for the design and optimization of automated driving systems.

## Figures and Tables

**Table 1:** Parameter definition table.

Parameters	Unit	Category	Definition
Steering wheel reversal rate	Counts/min	Lateral parameters	The times of the steering wheel turned per minute.
Maximum steering angle	Scale from 0–1	Lateral parameters	The maximum range of steering wheel turning.
Lateral deviation	meter	Lateral parameters	The maximum distance that the center of the vehicle deviated from the centerline of the initial lane.
Lateral deviation rate	m/s	Lateral parameters	The ratio of vehicle lateral deviation to take-over time with this phase.
Average speed	Km/h	Longitudinal parameters	The average value of the speed during the take-over process.
Braking rate	Counts/min	Longitudinal parameters	Number of brake pedal operations per minute.
Accelerating rate	Counts/min	Longitudinal parameters	Number of acceleration pedal operations per minute.
Reaction time	Seconds	Take-over timing	The interval between the start of the take-over signal and the driver’s switch moment by pressing the take-over button.
Control time	Seconds	Take-over timing	The interval between the start of the driver’s switch moment by pressing the take-over button and the moment vehicle completely moved inside the initial lane
Recovery time	Seconds	Take-over timing	The interval between the moment vehicle completely moved inside the initial lane and the moment the driver switched back to automated driving.

**Table 2:** Influence of take-over budget time and traffic density on lateral operation parameters.

Parameters	Phase	Influence Factors	F	*p*
Steering wheel reversal rate	Phase 2	Take-over budget time	6.767	0.013
		Traffic density	8.354	0.006
		Interaction effect	4.493	0.040
	Phase 3	Take-over budget time	2.631	0.113
		Traffic density	7.310	0.010
		Interaction effect	0.812	0.373
Maximum steering angle	Phase 2	Take-over budget time	0.439	0.511
		Traffic density	1.756	0.193
		Interaction effect	3.95	0.054
	Phase 3	Take-over budget time	0.914	0.345
		Traffic density	2.687	0.109
		Interaction effect	0.004	0.945

**Table 3:** Influence of take-over budget time and traffic density on lateral dynamics parameters.

Parameters	Phase	Influence Factors	F	*p*
Lateral deviation	Phase 2	Take-over budget time	2.172	0.149
		Traffic density	0.035	0.851
		Interaction effect	5.708	0.022
	Phase 3	Take-over budget time	0.007	0.932
		Traffic density	0.889	0.351
		Interaction effect	0.172	0.680
Lateral deviation rate	Phase 2	Take-over budget time	7.710	0.008
		Traffic density	23.269	<0.001
		Interaction effect	2.850	0.100
	Phase 3	Take-over budget time	3.466	0.070
		Traffic density	1.711	0.199
		Interaction effect	0.136	0.714

**Table 4:** Influence of take-over budget time and traffic density on longitudinal dynamics parameters.

Parameters	Phase	Influence Factors	F	*p*
Average speed	Phase 2	Take-over budget time	25.6188	<0.001
		Traffic density	24.86789	<0.001
		Interaction effect	1.487468	0.230
	Phase 3	Take-over budget time	0.013185	0.909
		Traffic density	15.76549	<0.001
		Interaction effect	1.061875	0.309

**Table 5:** Influence of take-over budget time and traffic density on longitudinal operation parameters.

Parameters	Phase	Influence Factors	F	*p*
Accelerating rate	Phase 2	Take-over budget time	0.521	0.474
		Traffic density	8.347	0.006
		Interaction effect	<0.001	<0.001
	Phase 3	Take-over budget time	21.887	<0.001
		Traffic density	18.525	<0.001
		Interaction effect	0.315	0.578
Braking rate	Phase 2	Take-over budget time	34.56	<0.001
		Traffic density	15.36	<0.001
		Interaction effect	0.24	0.627
	Phase 3	Take-over budget time	1.975	0.168
		Traffic density	1.975	0.168
		Interaction effect	10.756	0.002

**Table 6:** Influence of take-over budget time and traffic density on take-over time.

Parameters	Phase	Influence Factors	F	*p*
Take-over time	Phase 1	Take-over budget time	24.694	<0.001
		Traffic density	3.717	0.061
		Interaction effect	0.006	0.935
	Phase 2	Take-over budget time	5.437	0.025
		Traffic density	33.415	<0.001
		Interaction effect	0.102	0.750
	Phase 3	Take-over budget time	25.018	<0.001
		Traffic density	27.279	<0.001
		Interaction effect	1.642	0.208

## Data Availability

Data will be made available on reasonable request from the corresponding author.
